# Effects of Jiawei Shaoyao-Gancao Decoction and Its Drug-Containing Serum on Proliferation, Apoptosis, and Ultrastructure of Human Adenomyosis Foci Cells

**DOI:** 10.1155/2017/7821095

**Published:** 2017-09-12

**Authors:** Yu-yan Zeng, Kun-yin Li

**Affiliations:** ^1^Department of Gynecology, The Second Affiliated Hospital of Guangzhou University of Chinese Medicine, Guangzhou 510120, China; ^2^Postdoctoral Programme, Guangzhou University of Chinese Medicine, Guangzhou 510006, China; ^3^Guangzhou University of Chinese Medicine, Guangzhou 510006, China

## Abstract

**Objective:**

The present study aimed to investigate the effects of Jiawei Shaoyao-Gancao Decoction (JSGD) and its drug-containing serum (CDS) on the proliferation, apoptosis, and ultrastructure of human adenomyosis foci cells.

**Methods:**

Primary cultures of human adenomyosis foci cells were prepared from hard uterine lesions of adenomyosis patients. The cells were treated with JSGD (10 and 20 mg/ml), CDS, and mifepristone (MIF) for 24 or 48 h. Cell proliferation was detected using CCK-8 assay, cell apoptosis was measured by flow cytometry, and the cell ultrastructure was observed by transmission electron microscopy (TEM).

**Results:**

JSGD and CDS significantly induced cell apoptosis and inhibited cell proliferation for 24 h or 48 h, in which the effects of JSGD were in a dose-dependent manner. The effect of CDS for 24 h was higher than that of CDS for 48 h. Moreover, JSGD and CDS treatments induced marked apoptosis in adenomyosis foci cells, characterized by nucleus chromatin, condensation, fragmentation, mitochondria and endoplasmic swelling, and autophagy-lysosome.

**Conclusions:**

JSGD and CDS can suppress proliferation and induce apoptosis in adenomyosis foci cells, through altering their ultrastructure. The results provided support for JSGD and CDS in the treatment of adenomyosis and gained further insight into the effect of this prescription.

## 1. Introduction

Adenomyosis is a gynecological benign tumor that refers to endometrial glands or stroma penetrating the endometrial surface, diffusely or locally infiltrating into myometrium, compensatory hypertrophy, and hyperplasia of peripheral muscular cells. Although the disease is benign, it can cause many health problems for women, such as dysmenorrhea, menorrhagia, early pregnancy-stage miscarriages, and even infertility with a prevalence of 70% and dysmenorrhea rate of 77% [1–3]. Patients' general health perception and body and social function with adenomyosis dysmenorrhea are all lower than those of healthy people [4]. The main drug therapy for young women with fertility requirements is gonadotrophin-releasing hormone (GnRH). However, its potential side effects and drug withdrawal symptoms compromise its clinical application [5]. Hysterectomy is a kind of radical cure, but it is not suitable for patients with fertility requirements. Conservative surgery may retain fertility; however, the disease easily recurs. And surgical treatment for patients causes physical and mental trauma. Thus, novel therapeutic strategies are urgently needed and traditional Chinese medicine is deemed promising to improve the clinical management of patients with adenomyosis.

Shaoyao-Gancao Decoction (SGD) is a famous traditional Chinese medicine prescription used for the treatment of adenomyosis and dysmenorrheal and abdominal pain, which is sourced from the Chinese Medical Classics—*Shanghan Lun* in 210 CE. SGD consists of Paeoniae Radix (Shao-Yao in Chinese) and Glycyrrhizae Radix (Gan-Cao). Huayu-Zhitong Decoction (HZD) was proposed by Professor Kun-yin Li, with rich experience in adenomyosis treatment, and Professor Hui-qing Ou-yang, one of the third group of National Famous Old Doctor of traditional Chinese medicine in China. HZD has been applied in the clinic for more than 10 years and proved to have pronounced efficacy in alleviating dysmenorrhea rate by about 90% [6]. Thus, we optimized and proposed a Jiawei Shaoyao-Gancao Decoction (JSGD, composed of Paeoniae Radix (Shao-Yao), Glycyrrhizae Radix (Gan-Cao), Panax Notoginseng (San-Qi), Radix Angelicae Sinensis (Dang-Gui), and Rhizoma Corydalis (Yanhu-Suo)) on the basis of former clinical and experimental studies [6–8] and the foundation of classic prescription “SGD” and experience prescription “HZD.” In this study, we aimed to investigate the potential effects of JSGD and CDS on the proliferation, apoptosis, and ultrastructure of human adenomyosis foci cells* in vitro*.

## 2. Materials and Methods

### 2.1. Samples Selected

Six cases of adenomyosis patients aged 20–50 years were selected in the First Affiliated Hospital of Guangzhou University of Chinese Medicine between September 2014 and January 2015 with surgical treatment. No hormone or similar drugs were used for 6 months before treatment. All cases were confirmed by pathological results.

### 2.2. Method of Specimens

Under sterile conditions in the operating room, hard uterine lesions were cut out to soak in precooled PBS and sent to the laboratory within half an hour; those of the pathological results after operation that did not accord with the diagnosis criteria were timely eliminated.

### 2.3. Reagent and Instrument

Annexin V-FITC Apoptosis Detection Kit (catalog number FP617) and Cell Counting Kit-8 (CCK-8, catalog number GC763) (Dojindo Ltd., Japan), high glucose DMEM (catalog number 81150053), 0.25% EDTA-free Trypsin (catalog number 1394362), and fetal bovine serum (catalog number 41F3743K) were obtained from Gibco. Collagenase type I (catalog number 17100-017) was purchased from Invitrogen. Mouse anti-human actin, vimentin, and broad-spectrum cytokeratin monoclonal antibody kit (batches numbers ZM-0003, ZM-0260, and ZM-0060, resp.), a two-step immunohistochemical detection kit for mouse, and rabbit hypersensitivity kit (batch number PV-9001) were obtained from Beijing Zhongshan Jinqiao Biological Technology. A transmission electron microscope (TEM) of fixed liquid was provided at the electron microscope room of Zhongshan University. Mifepristone (national drug approval number H10950003) was purchased from Beijing Zizhu Pharmaceutical Ltd. A cell incubator of constant temperature CO_2_ (37°C, 5% CO_2_) was provided by American Harris Corporation. An automatic inverted microscope (ix70) was provided by Olympus Company. Flow cytometry instrument was provided by the American Beckman Coulter Inc. Multiskan MK3 ELISA was provided by Thermo Company. And a transmission electron microscope was provided by American FEI Inc., model Tecnai G^2^ Spirit Twin.

### 2.4. Drug Preparation and Grouping

#### 2.4.1. JSGD Water Extract Preparation

Chinese herbal medicines were purchased according to the original prescription and prepared and stored according to our previous methods [9]. JSGD high concentration was 20 mg/ml and JSGD low concentration was 10 mg/ml, which were ready to use.

#### 2.4.2. Positive Control Group

An appropriate amount of mifepristone (25 mg/tablet) was weighed to prepare 5 × 10^−2^ mol/L in DMSO and diluted to 1 × 10^−5^ mol/L (guaranteed final concentration of DMSO < 0.5%) on standby.

#### 2.4.3. Blank Control Group

High glucose DMEM with no serum was used.

#### 2.4.4. Preparation of CDS

*(1) Experimental Animals*. Ten female rats of SPF (body weight 200 ± 20 g, animal certificate number 44007200013883, license: scxk-0002; certificate of feed: 44200300002496, license: scxk (Guangdong) 2013-0002) were all provided by Guangdong Experimental Animal Center: 3 rats for blank serum group and 7 rats for CDS group.

*(2) The Dosage and Preparation*. JSGD rats were given 2.31 × 10^3^ mg/kg by intragastric administration according to 5 times the amount of the equivalent dose conversion coefficient of standard animals of “Methodology of Pharmacological Experiment” [10]. Blank serum group rats were given physiological saline (2.31 × 10^3^ mg/kg) by intragastric administration. Drug administration time was at 10:00 a.m. and 10:00 p.m. Water but not feed was provided on the third day; rats were intraperitoneally anesthetized by 10% chloral hydrate after 1 h for the last lavage. Then, blood was taken from the abdominal aorta, centrifuged, in a water bath, disinfected with a filter membrane, and preserved in a −80°C refrigerator, avoiding repeated freezing and thawing.

*(3) Including Medicine Culture Preparation and Grouping*

*CDS Group*. We screened out 10% of the drug-containing serum through preliminary experiments by the CCK-8 assay, which was made up of 10 percent drug-containing serum and 90 percent high glucose and stored in a 4°C refrigerator on standby.

*Blank Serum Group*. It was made up of 10 percent blank serum and 90 percent high glucose and stored in a 4°C refrigerator on standby.

### 2.5. Cultivation, Passage, and Identification of Cells

The specimens were digested by collagenase and for the primary cell culture with 1% double antibiotic and 10% fetal bovine serum. Cells were passaged every 5–7 days and the third- to seventh-generation cells were used for the study. The first-generation cells were identified by immunocytochemistry.

### 2.6. Effect of JSGD and CDS on Cell Proliferation, Apoptosis, and Ultrastructure

#### 2.6.1. CCK-8 Assay of Cell Proliferative Inhibition Rate

According to preliminary experiments, density and reaction time of the fourth-generation cells were determined: 6 × 10^5^ cells/ml and incubation for 1 h in 96-well plates. Each group had an experimental well, contrast well (including cell medium and CCK-8 reagent, not drug), and blank well (including the medium except for the cells and drugs, in addition to the CCK-8 reagent). Cell proliferative inhibition rate = (OD value of contrast well − OD value of experimental well)/(OD value of contrast well − OD value of blank well) × 100%.

#### 2.6.2. Flow Cytometric Analysis of Cell Apoptosis

The fourth-generation cells were evenly inoculated into 6-well plates and continued to be cultured for 24 h with high glucose DMEM after the bottom was filled with cells, and then the supernatant was discarded after using drugs for 24 h or 48 h. Cells were digested for 6 to 8 min by 0.25% EDTA-free Trypsin, FBS medium was added, and the wall of the well was gently blown to stop digestion, followed by centrifugation; then, the supernatant was discarded, precooled PBS wash was added, followed by centrifugation twice, and then 1× Annexin V Binding Solution was added dropwise, prepared in advance to make a 1 × 10^6^ cells/ml cell suspension. 100 *μ*l cell suspension, 5 *μ*l Annexin V-FITC conjugate, and 5 *μ*l PI solution were put in turn into tubes, which were cultured for 15 min at room temperature. 400 *μ*l of 1× Annexin V Binding solution was added in each tube and detected in 1 h.

#### 2.6.3. Ultrastructural Observation by TEM

The abandoned supernatant of the fourth-generation cells joined the former fixed liquid. Cells were gently scraped, followed by centrifugation, and the supernatant was discarded and poured slowly into a prefixed fluid with fixed liquid containing 2.5% glutaric aldehyde, 2% paraformaldehyde, and 0.1% phosphate buffer fixed. PBS was washed 3 times, fixed with 1% osmic acid and 0.1 mol/L phosphate buffer, then dehydrated with 50%, 70%, 90%, and 100% ethanol dehydration step by step, dehydrated again with acetone, paraffined and sectioned, framed in a copper net, stained with uranium acetate and lead citrate, and finally observed and photographed by TEM.

### 2.7. Statistical Approach

SAS9.0 software was used for data processing. Levene tested homogeneity of variance; if the data followed normal distribution and homogeneity of variance, variance analysis was used. When the total difference was significant, the LSD method was used for pairwise comparison. If the data did not follow the homogeneity of variance, after the Wilcoxon score, the Kruskal-Wallis test was carried out. All results with *P* < 0.05 were defined as statistically significant.

## 3. Results

### 3.1. Identification of Cells

**Figure 1:**
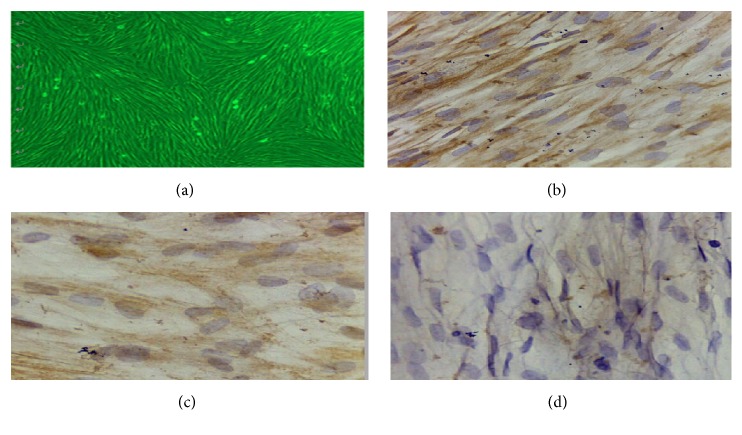
Identification of adenomyosis foci cells. (a) Observation of uterine adenomyosis cells cultured for 72 h under an inverted microscope. (b) Positive expression of keratin (×400). (c) Positive expression of vimentin (×400). (d) Positive expression of smooth muscle protein (×400).

All the 6 cases of cells were successfully cultured. The cultured cells with smooth muscle protein, vimentin, and keratin positive expression were identified as adenomyosis cells, as shown in Figure 1.

### 3.2. Effect of JSGD and CDS on Cell Proliferative Inhibition Rate

**Figure 2:**
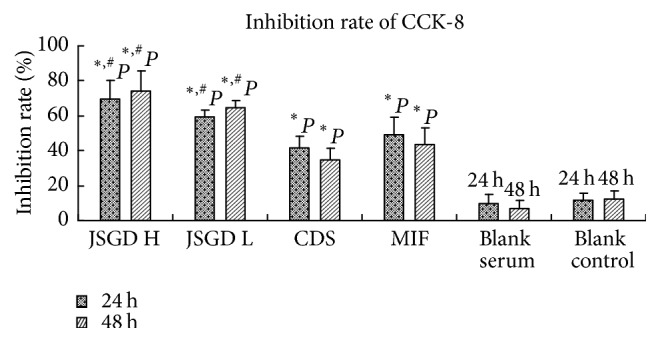
JSGD high concentration (JSGD H), JSGD low concentration (JSGD L), CDS, MIF, blank serum, and blank control; treatment results on inhibition rate of human adenomyosis foci cells. Inhibition rate was assessed by CCK-8. MIF and blank serum were used as a positive control group. The data were expressed as mean ± SD. ^*∗*^*P* < 0.05 indicated the groups with a significant difference when compared to blank serum and blank control groups. ^#^*P* < 0.05 indicated the groups of JSGD high concentration and JSGD low concentration with a significant difference when compared to the other groups.

Cell proliferative inhibition rate of JSGD high concentration for 48 h group was statistically higher than that of the other groups (*P* < 0.05), except for JSGD high concentration for 24 h group (*P* > 0.05). Compared with the blank control group and blank serum group, the inhibition rate of the remaining groups increased (*P* < 0.05). JSGD high concentration group (including drug for 24 h and 48 h groups) had the greatest inhibitory effect on the cells of adenomyosis, followed by JSGD low concentration group (including drug for 24 h and 48 h groups); the effect was concentration dependent, with no obvious correlation with the intervention time point. CDS and MIF groups could significantly inhibit the cell proliferation; the effect was worse than JSGD (including high and low concentration, drug for 24 h and 48 h groups), as shown in Table 1 and Figure 2.

### 3.3. Effect of JSGD and CDS on Cell Apoptosis Rate

**Figure 3:**
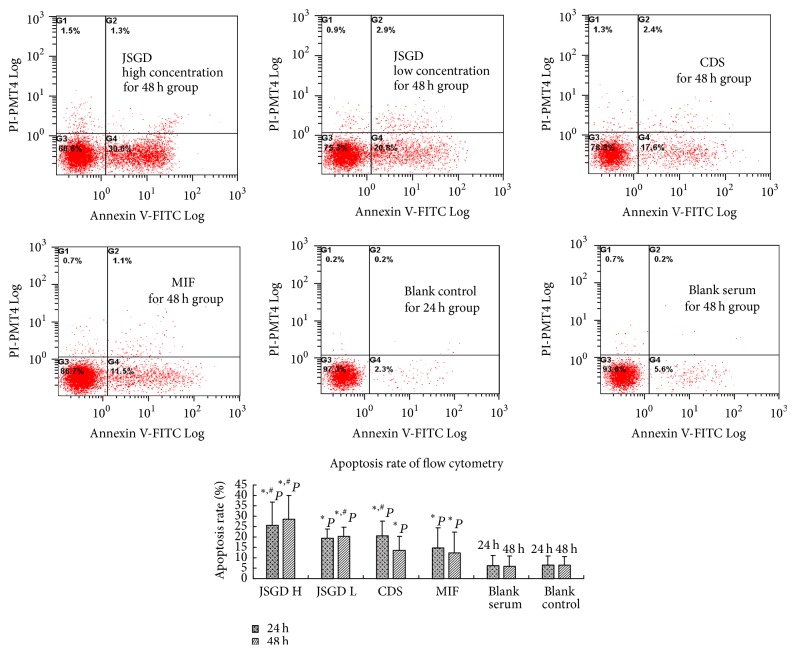
JSGD high concentration (JSGD H) for 48 h, JSGD low concentration (JSGD L) for 48 h, CDS for 48 h, MIF for 48 h, blank control for 24 h, and blank serum for 48 h; treatment results on apoptosis of human adenomyosis foci cells. Apoptotic cells were assessed by Annexin V-FITC and PI staining. MIF and blank serum were used as a positive control group. The data were expressed as mean ± SD. ^*∗*^*P* < 0.05 and ^#^*P* < 0.05 indicated the groups with a significant difference when compared to the other groups.

Cell apoptosis rate of JSGD high concentration for 48 h group was statistically higher than that of the other groups (*P* < 0.05), except for JSGD high concentration for 24 h group (*P* > 0.05). Compared with the blank control group and blank serum group, apoptosis rate of the remaining groups increased (*P* < 0.05). JSGD high concentration group (including drug for 24 h and 48 h) had the greatest effect on the cell apoptosis of adenomyosis, followed by JSGD low concentration group (including drug for 24 h and 48 h); the effect was concentration dependent, with no obvious correlation with the intervention time point. The cell apoptosis rate of CDS for 24 h group was significantly higher than that of CDS for 48 h group and MIF group (including drug for 24 h and 48 h) (*P* < 0.05), as shown in Figure 3 and Table 2.

### 3.4. Effect of JSGD and CDS on the Cell Ultrastructure

**Figure 4:**
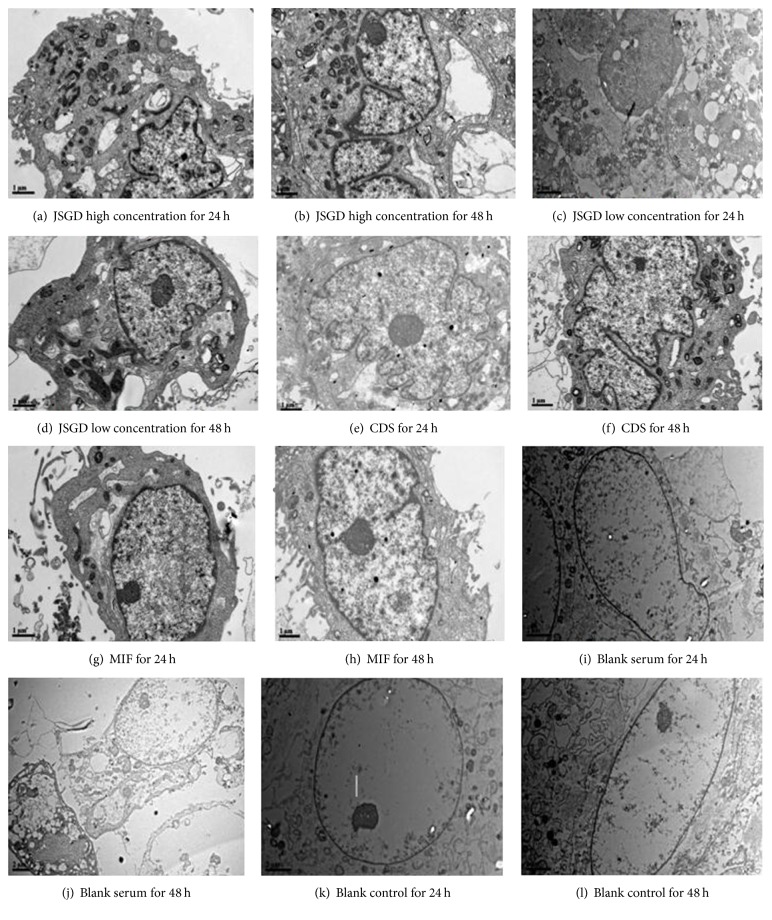
Effect of drug intervention on cell ultrastructure. Treatment results on the cell ultrastructure of human adenomyosis foci cells were observed by TEM. MIF and blank serum were used as a positive control group. The figures showed that the early apoptosis phenomenon appeared with nuclear chromatin condensed, chromatin edge moved, mitochondria swelling, endoplasmic reticulum expanded, and lysosomes of cytoplasm turned up, and even autophagy-lysosome for all other groups except the blank serum and blank control groups.

The chromatin of JSGD low concentration for 24 h group split into a number of pieces interspersing the nucleoplasm condensation; the nuclear membrane and cytoplasm membrane showed integrity, and organelles were indistinct; there were a large number of lysosomes in the cytoplasm and the formation of vacuoles was observed. The nucleolus of JSGD low concentration for 48 h group enlarged, nuclear chromatin moved, cells condensed, mitochondria showed swelling, and the endoplasmic reticulum expanded; the cytoplasmic membrane and nuclear membrane showed integrity, and there were lysosomes in the cytoplasm. The nucleolus of JSGD low concentration group (including drug for 24 h and 48 h) was cataclastic. Nuclear chromatin moved, condensed, and showed an irregular shape. The cytoplasm membrane and organelles showed integrity, and mitochondria showed swelling. There were a large number of lysosomes in the cytoplasm and part of the vacuole formation and autophagy bubble structure of bilayer and multilayer structure appeared, and even the autophagy structure was observed. Cells of CDS group (including drug for 24 h and 48 h) showed an irregular shape. Cells and nucleoplasm showed shrinkage and density increased, and the nuclear membrane and cytoplasm membrane showed integrity. Mitochondria showed swelling and the endoplasmic reticulum showed slight dilatation. There were many lysosomes in the cytoplasm. Cells and nucleoplasm of the MIF group (including drug for 24 h and 48 h) showed shrinkage, the nucleolus moved, and mitochondria showed swelling. There were many lysosomes in the cytoplasm and formation of vacuoles. There were more autosomes in cells of the blank serum and blank control groups (including drug for 24 h and 48 h); the nuclear membrane, cytoplasm membrane, and organelles showed integrity, the cell surface was plump round with no apparent bump, cells were without enrichment, mitochondria showed mild swelling, and there was a small amount of lysosomes in the cytoplasm, as shown in Figure 4.

## 4. Discussion

Cell proliferation is the process of cells under the effect of related factors to supplement the aging or dying cells* in vivo* by cell division. Apoptosis is an orderly process of activating the death program stored in the cells after receiving certain factors [11]. Apoptosis occurs in 3 stages: (1) finalize the design phase: receiving the death signal; (2) implementation period: cells shrink and mitochondria and ribosomes gather themselves together and form an apoptotic body; (3) phagocytic period: apoptotic bodies are consumed and apoptosis is completed. The three phases are coherent and indivisible, and the morphology of cell apoptosis presents different characteristics according to different periods.

### 4.1. Effect of JSGD and CDS on Proliferative Inhibition Rate and Ultrastructure of Adenomyosis Foci Cells

Research shows that the new cell activity detection method CCK-8 has the advantages of high stability and sensitivity, simplified operation steps to save time, and low toxicity [12]. This study detected proliferative inhibition rate after drug intervention of adenomyosis foci cells by CCK-8 assay. Results showed that JSGD, CDS, and MIF could inhibit proliferation of adenomyosis foci cells, in which the effect of JSGD was concentration dependent, with the strongest effect of high concentration group.

Based on confirmation of cell proliferative inhibition, we viewed the cell ultrastructure under TEM after drug intervention. Results showed that the early apoptosis phenomena appeared with nuclear chromatin condensed, chromatin edge moved, mitochondria swelling, and endoplasmic reticulum expanded. Lysosomes of the cytoplasm appeared and even autophagy-lysosome turned up for all other groups except for the blank serum and blank control groups. As we know, the endoplasmic reticulum is the key to ensure the cell activity and survival, and mitochondria are not only the respiratory chain and oxidation center of cells, but also the cell apoptosis control center. The Mitochondrial pathway of apoptosis functions in response to various types of intracellular stress and results in the increase of mitochondrial membrane permeability and activation of apoptosis-related proteins to induce apoptosis [13]. We guess  that JSGD and CDS could start the endoplasmic reticulum and mitochondrial apoptosis system to inhibit cell proliferation and promote apoptosis by changing the structure of the endoplasmic reticulum, mitochondria, and other organelles [14, 15]. Lysosome is a kind of vesicle surrounded by a single membrane with acid hydrolase, which plays an important role in material circulation and physiological processes. Studies have shown that [16] lysosomes can induce cell apoptosis. Lysosomes, influenced by a variety of apoptotic factors, could result in changes of lysosome membrane permeability and induce cathepsin B and cathepsin L release, which through changing the balance of iron in cells and the expression level of reactive oxygen species induces apoptosis [17]. Autophagy is a stress response that degrades cell components such as damage and aging in order to maintain the cell homeostasis, which combines with the lysosome as autophagy-lysosome. The relationship between autophagy and apoptosis is a double-edged sword, which both can be synergistic and antagonistic. Referring to the results of this study, we think that JSGD and CDS may induce apoptosis by increasing the number of lysosomes in the cytoplasm, which combine with autophagy resulting in a series of chemical changes.

### 4.2. Effect of JSGD and CDS on Apoptosis Rate of Adenomyosis Foci Cells

Based on the apoptosis phenomenon under TEM, we further used flow cytometry Annexin V-FITC/PI staining detecting changes in the rate of apoptosis after drug intervention. Results showed that the effects of JSGD for 24 h and 48 h group and CDS for 24 h group were better than those of MIF. The effect of JSGD was concentration dependent, with no obvious correlation with the intervention time point. Once again, JSGD was proved to play an important role in the treatment of adenomyosis by promoting cell apoptosis.

### 4.3. Comparison of the Effects of JSGD and CDS on Adenomyosis Foci Cells

Many researchers believe that [18] the effective component in Chinese medicine is complex, and many components do not function until they undergo a series of biotransformations* in vivo*. The product of herbs has a biological activity after flora metabolism; thus, they put forward the “traditional Chinese medicine serum pharmacology” through the effect of herbs* in vivo* to collect blood and pass a series of technical treatment methods. The method can both overcome the interference of herbs' factors such as physical and chemical properties and simulate objectively the interaction process between drugs and the body and can further reflect the pharmacological effects of herbs' parent drug and their metabolites, making the experiment more scientific and authentic.

Results of this study showed that JSGD and CDS could obviously inhibit proliferation and promote apoptosis of adenomyosis foci cells, which suggest that the effects of herbal compounds and herbal compounds drug-containing serum are consistent, and the two methods of drug extraction prove that JSGD is effective in the treatment of adenomyosis.

## 5. Conclusions

In conclusion, we cultured primary cells by collecting directly hard uterine lesions of adenomyosis patients as experimental subjects to reveal the effects of JSGD and CDS on adenomyosis foci cells through different concentrations and different time points, which was more scientific and realistic than an animal model. Moreover, we verified that JSGD and CDS could inhibit cell proliferation by the CCK-8 method and induce early apoptosis of cell ultrastructure under TEM. And further we used the Annexin V-FITC/PI double staining to detect changes in the apoptosis rate after drug intervention and affirmed that the effects of JSGD were the highest. We finally confirmed that JSGD inhibited proliferation and promoted apoptosis of adenomyosis foci cells by two kinds of drug extraction methods, traditional Chinese medicine compound water extraction liquid and animal serum drug, in which the effects were concentration dependent, with no obvious correlation with the intervention time. As for the JSGD, how to promote cell apoptosis, whether it is associated with a series of changes such as activating the apoptosis pathway of endoplasmic reticulum and mitochondria, changing the permeability of lysosomal membrane, and inducing autophagy are still not clear. The specific signal mechanism and targets need further discussion.

## Figures and Tables

**Table 1:** Effect of JSGD and CDS on inhibition rate of adenomyosis foci cells (*N* = 6, mean ± SD).

Group	Cell proliferative inhibition rate (%)	
	*T* = 24 h	*T* = 48 h
JSGD high concentration	69.24 ± 9.61^*∗*^	74.48 ± 6.53^*∗*^
JSGD low concentration	58.93 ± 7.20^*∗*^	64.32 ± 7.62^*∗*^
CDS	41.24 ± 11.24^*∗*^	34.51 ± 17.29^*∗*^
MIF	48.92 ± 15.35^*∗*^	43.33 ± 5.16^*∗*^
Blank serum	9.82 ± 4.56	6.82 ± 4.00
Blank control	11.52 ± 3.03	12.38 ± 3.02

^*∗*^ *P* < 0.05, compared with blank serum and blank control group.

**Table 2:** Effect of JSGD and CDS on apoptosis rate of adenomyosis foci cells (*N* = 6, mean ± SD).

Group	Cell apoptosis rate (%)	
	*T* = 24 h	*T* = 48 h
JSGD high concentration	25.65 ± 4.49^*∗*#^	28.63 ± 4.01^*∗*#^
JSGD low concentration	19.32 ± 5.21^*∗*&^	20.18 ± 5.33^*∗*#&^
CDS	20.68 ± 8.32^*∗*#&^	13.40 ± 6.49^*∗*&^
MIF	14.57 ± 4.70^*∗*^	12.48 ± 6.01^*∗*^
Blank serum	6.17 ± 1.83	5.88 ± 3.14
Blank control	6.55 ± 3.73	6.38 ± 3.29

^*∗*^ *P* < 0.05, compared with blank control for 24 h and 48 h group; ^#^*P* < 0.05, compared with MIF for 24 h and 48 h group; ^&^*P* < 0.05, compared with JSGD high concentration for 48 h group.
